# Screening opportunistic osteoporosis through multimodal techniques of hip joint CT images: exploring 2D and 3D deep learning, radiomics, clinical data, and their integration

**DOI:** 10.3389/fendo.2026.1838830

**Published:** 2026-07-17

**Authors:** Xiaocong Lin, Xiaoling Zheng, Shaojian Shi, Rongkai Shen, Kaibin Fang

**Affiliations:** 1 Department of Sports Medicine, The Second Affiliated Hospital of Fujian Medical University, Quanzhou, China; 2 Liming Vocational University, Quanzhou, China; 3 Department of Orthopaedic Surgery, Jinjiang General Hospital, Quanzhou, China; 4 Department of Orthopaedic Surgery, The First Affiliated Hospital of Fujian Medical University, Fuzhou, China

**Keywords:** computed tomography, deep learning, osteoporosis, radiomics, region of interest

## Abstract

**Background:**

Osteoporosis is a systemic disorder that is characterized by reduced bone mass and density, resulting in heightened bone fragility and vulnerability to fractures. The opportunistic screening of osteoporosis through hip CT holds significant clinical significance.

**Purpose:**

This study explores methods for osteoporosis detection using hip CT data analysis, integrating 2D/3D deep learning, radiomics, and clinical data to improve diagnostic accuracy and reliability.

**Methods:**

This study enrolled a total of 567 patients with hip joint CT images. Clinical data, including age, gender, and other relevant factors, were utilized to establish an opportunistic osteoporosis screening clinical model. Regions of interest on the patients’ hip joint CT scans were outlined and assessed for opportunistic osteoporosis screening through radiomic techniques, 2D deep learning technology, and 3D deep learning technology. A Nomogram model for opportunistic osteoporosis screening based on hip joint CT was established after integrating the radiomic model with the clinical model. The efficacy of each model was compared to identify the optimal model for opportunistic osteoporosis screening based on hip joint CT.

**Results:**

Among all Nomogram models (radiomics model + clinical model), the GradientBoosting machine learning algorithm demonstrated the best performance in the validation group. The accuracy of this model in screening for opportunistic osteoporosis in the validation group was 0.849, with an AUC of 0.911. Among all two-dimensional deep learning models, densenet201 exhibited the best performance in the validation group. The accuracy of this model in screening for opportunistic osteoporosis in the validation group was 0.817, with an AUC of 0.884. In three-dimensional deep learning models, the three-dimensional ResNet34 showed the best performance in the validation group, with an accuracy of 0.806 and an AUC of 0.889.

**Conclusions:**

Our study showcases the potential of employing radiomics, Nomogram techniques and deep learning techniques to analyze CT images of patients’ hips, facilitating the evaluation of osteoporosis.

## Introduction

Osteoporosis is a systemic disorder that is characterized by reduced bone mass and density, resulting in heightened bone fragility and vulnerability to fractures (1). With the escalating phenomenon of population aging, osteoporosis and its associated complications, such as fractures, have emerged as critical public health challenges (2). Research has demonstrated that the prevalence of osteoporosis in China varies between 6.6% and 19.3%, with standardized age-specific prevalence rates of approximately 10% for males and 30% for females aged 50 and above (3). At present, there are over 10 million male patients and more than 40 million female patients suffering from osteoporosis in China (4). The same burden of disease is observed in other countries as well (5, 6). Despite the high incidence rate of osteoporosis, effective treatment plans and prevention strategies enable the diagnosis and management of this disease (7). Consequently, early screening and detection of osteoporosis hold significant importance (8).

In the realm of osteoporosis, the provision of timely screening and treatment remains an elusive goal for a significant proportion of patients (9). Regrettably, merely one-third of eligible patients have availed themselves of dual-energy X-ray absorptiometry (DXA) testing (10). Given the insidious nature of osteoporosis, which typically remains asymptomatic until a fracture occurs, this diagnostic and treatment rate falls far short of satisfying expectations (11). Numerous individuals only undergo DXA assessments subsequent to experiencing a fracture, in an attempt to ascertain the presence of osteoporosis. However, it is worth noting that patients who have already sustained fractures and then undergo additional DXA evaluations often endure heightened levels of discomfort, particularly when these assessments are performed prior to surgery. One promising approach to ameliorate this scenario involves the implementation of computed tomography (CT) scans as a component of routine outpatient care, enabling “opportunistic” screening for osteoporosis without incurring additional expenses or subjecting patients to excessive radiation (12). Furthermore, in instances where patients have already encountered hip injuries, often necessitating a thorough diagnosis or comprehension of the fracture dynamics, it is common practice for patients to undergo hip CT examinations (13). By conducting preliminary osteoporosis screenings via hip CT, the potential reduction in both costs and radiation exposure emerges as a pivotal factor of paramount importance in the diagnosis and treatment of patients.

In recent years, the realm of osteoporosis research has witnessed the advent of artificial intelligence (AI) and its burgeoning applications, particularly in the domains of radiomics (14) and deep learning (15). Radiomics, a swiftly advancing discipline, encompasses the extraction of quantitative indicators or radiological features from medical images, thereby enabling the use of intricate data modeling algorithms to elucidate image attributes beyond conventional visual detection (16). Specifically, CT-based radiomics features hold tremendous potential for facilitating diagnosis without incurring supplementary medical costs or subjecting patients to unnecessary radiation exposure. Deep learning represents a pivotal area of investigation. In conjunction with its proficiency in image classification, extracting deep learning features from neural networks has emerged as a captivating research focus (17). While X-ray is a commonly used modality in medical imaging, most imaging techniques, such as CT and MRI, capture three-dimensional (3D) images of the human body. Typically, for 3D images, the section with the largest cross-sectional area is extracted and utilized for deep learning or other subsequent research purposes (18). Notably, research studies have demonstrated promising outcomes from the application of 3D image analysis using deep learning techniques to identify various diseases (19). In this study, our aim is to seamlessly incorporate the emerging fields of radiomics and deep learning in order to leverage their potential in extracting crucial features from CT images of the hip joint. Our ultimate objective is to advance the diagnosis of osteoporosis. Additionally, we will employ both 2D and 3D image-based deep learning techniques, while extracting fundamental clinical information of patients. By comparing and integrating this data, we will strive to derive the most effective diagnostic model for osteoporosis. Through the adoption of these approaches, we aspire not only to augment the detection rate of opportunistic osteoporosis, but also to alleviate the additional economic burden and radiation exposure that patients with hip joint injuries encounter.

To address the specific scientific and clinical questions underlying opportunistic osteoporosis screening using hip CT, we designed a multi−model comparative framework. First, the clinical model. With this model, we aimed to address whether simple demographic variables (age and sex) alone can achieve acceptable performance for opportunistic osteoporosis screening. Establishing this baseline allowed us to quantify the incremental value added by incorporating imaging features from CT scans. Second, the radiomics model. We sought to determine whether radiomics features extracted from the hip CT ROI provide additional discriminatory power beyond clinical variables. Radiomics captures subvisual tissue characteristics that may reflect trabecular microarchitecture alterations associated with osteoporosis. By comparing radiomics−based models against the clinical baseline, we aimed to assess whether CT image information content alone could improve diagnostic accuracy. Third, the nomogram model (radiomics + clinical). We investigated whether integrating radiomics features with demographic variables yields superior performance compared to either approach alone. The nomogram model tested whether the two information sources provide complementary rather than redundant predictive value. Fourth, the deep learning models (2D vs. 3D). The development of deep learning models primarily addressed two interconnected questions: (a) Can deep learning models automatically learn osteoporosis−relevant imaging features without manual feature extraction? (b) Does 3D volumetric analysis provide incremental benefit over 2D image−based analysis? While 2D deep learning using the largest cross−sectional area of the ROI is computationally efficient and commonly reported, 3D deep learning may capture volumetric bone mineral density distribution and spatial structural information that 2D approaches might miss. By comparing these two approaches, we aimed to evaluate the trade−off between computational cost and potential diagnostic gain. Fifth, inclusion of multiple machine learning algorithms within each modeling strategy. This was not intended as an exhaustive benchmark but rather to ensure that our conclusions about the relative value of each modeling strategy were not biased by the choice of a particular classifier. By identifying the best−performing algorithm for each strategy, we could make fair comparisons across strategies.

## Materials and methods

### Participants in the study and development of clinical models.

The study’s inclusion criteria are as follows: 1, Participants who underwent both hip computed tomography (CT) scans and dual-energy X-ray absorptiometry (DXA) bone density assessments within a one-month interval, spanning from January 2015 to December 2023. 2, Individuals aged 50 years or older. Exclusion parameters include: 1, Inability to define the region of interest (ROI) due to bilateral hip fractures or articular pathologies. 2, Presence of internal fixation devices causing artifacts that obstruct ROI definition.

**Figure 1:**
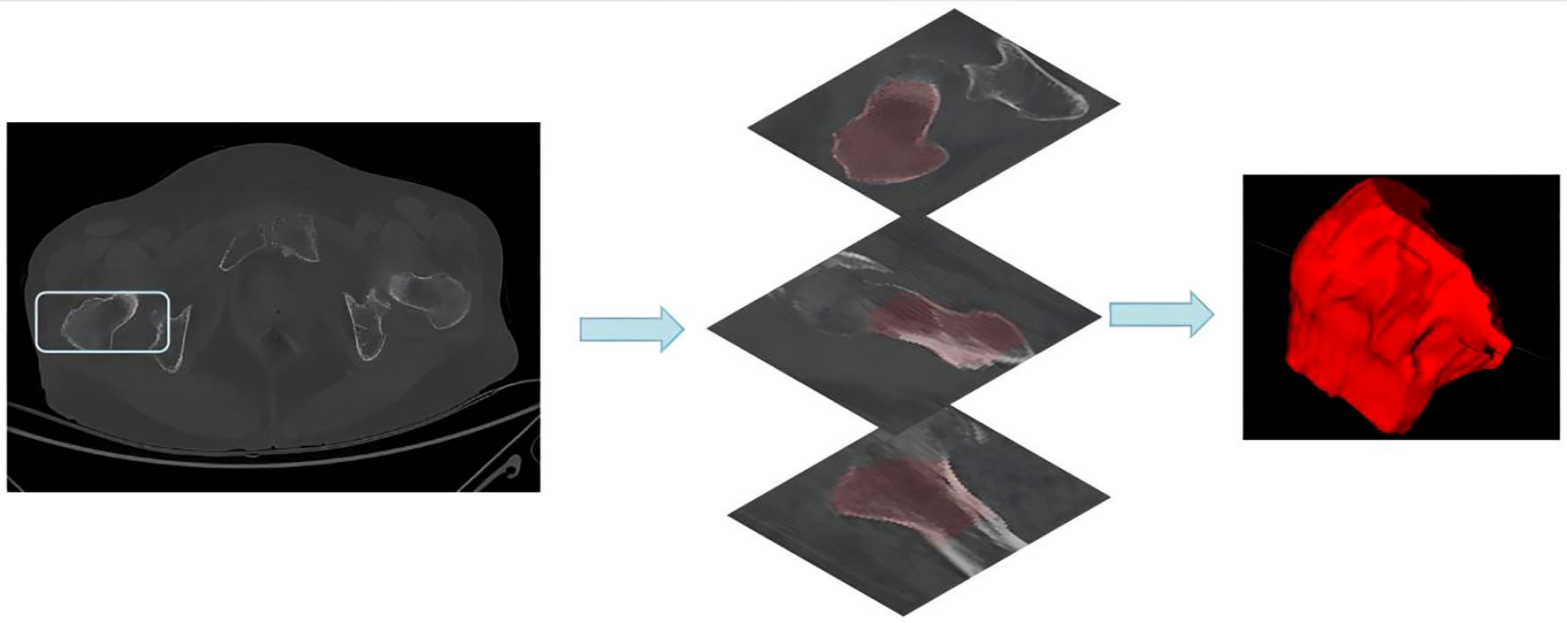
The process of drawing the region of interest (ROI).

In total, this retrospective investigation compiled hip CT scans alongside corresponding DXA outcomes for 567 patients. The diagnosis of osteoporosis was anchored to DXA test findings. Osteoporosis was defined based on dual-energy X-ray absorptiometry (DXA) T-scores. The lowest T-score among the lumbar spine (L1-L4) and hip sites (femoral neck, total hip, and trochanter) was used for diagnosis. A T-score ≤ -2.5 at any of these sites was considered diagnostic of osteoporosis, in accordance with the World Health Organization criteria. Patients were sourced from a major healthcare facility and were partitioned randomly into an 8:2 ratio for training and internal validation cohorts, respectively. An independent external validation group, comprising data from another institution, added robustness to the analysis, consisting of 381 individuals in the training set, 93 in the internal validation set, and an additional 93 in the external validation set. Ethical clearance was secured from the Hospital’s Institutional Review Board prior to study initiation, adhering strictly to the ethical guidelines set forth by the Declaration of Helsinki. A visual representation of the inclusion and exclusion criteria is provided in **Figure 1**. Notably, DXA remains the definitive diagnostic tool for osteoporosis, with a T-score threshold of -2.5 or less confirming its presence.

### Imaging acquisitions

The evaluation of bone density in the lumbar vertebrae and hip was conducted using the Lunar Prodigy dual-energy X-ray absorptiometry (DEXA) equipment manufactured by GE, USA. This advanced machine demonstrates a remarkable accuracy deviation of merely 0.68% and a minimal detectable change of 1.88%. To ensure precise measurements, the apparatus underwent systematic calibration procedures before each daily assessment.

The designated CT scanning plan necessitated patients to assume a supine position, with their arms raised above their head, whilst undergoing a spiral scan of the hip region. The scanning range extended from the anterior superior iliac spine to the middle and upper femur. The established scanning parameters included a tube voltage of 120 kV, a tube current of 315mAs, a layer thickness of 3mm, and a layer spacing of 3mm.

Prior to commencing the experiment, it is essential to preprocess all the images. This involves the adjustment of the window width and level for all images to ensure compatibility with the bone window. Furthermore, a standardization process will be implemented, where the layer thickness and spacing of the images will be modified to maintain a consistent and uniform representation throughout the dataset.

### Data sets

Demographic information of patients was obtained from a specialized registration and appointment system, whereas the PACS system was utilized to collect relevant imaging data. Comparative analyses of the patients’ clinical characteristics were performed using independent-samples t tests and chi-square tests. The baseline clinical characteristics of the patient cohort are presented in **Table 1**.

**Table 1:** The baseline clinical characteristics of patients.

	Training group - osteoporosis	Training group - no osteoporosis	Statistical value		P value	Internal testing group - osteoporosis		Internal testing group - no osteoporosis		Statistical value	P value
Age (years)	72.93 ± 12.34	64.82 ± 11.69	5.97		<0.01	71.64 ± 15.07		64.00 ± 10.61		2.39	0.02
Gender			22.49		<0.01					4.55	0.03
Male	75	61				21		15			
Female	192	53				45		12			
	External validation group - Osteoporosis			External validation group - no osteoporosis			Statistical value		P value			
Age (years)	69.81 ± 11.75		57.77 ± 13.58			4.42		<0.01		
Gender						6.09		0.01		
Male	16		16							
Female	46		15							

### Clinical signature

Patient’s basic information such as gender and age were used to construct the clinical model. All patients were recorded with these basic details prior to their examination registration. After collecting this information, the DXA test results of the patients were assessed to determine if they had osteoporosis. The clinical data of the training group patients were analyzed through univariate and multivariate analyses to identify associations between factors like gender and age with osteoporosis. Indicators with P<0.05 in both univariate and multivariate analyses were selected to establish the clinical model. Spearman regression was used to calculate the correlation between these indicators and osteoporosis-related factors, thereby establishing a clinical model for screening opportunistic osteoporosis.

### Establishment of radiomics models

For the purpose of analysis, axial images of hip CT scans were specifically chosen. The ITK SNAP software was employed to reconstruct the images and accurately delineate the regions of interest (ROI). The drawing of ROI is done manually. The sketched area includes the femoral neck, trochanter, WARD’s triangle, and intertrochanteric space. This is consistent with the area where DXA is used to measure hip bone density (18). The process of ROI drawing is illustrated in **Figure 1**.

### Selection of optimal model

The selection of the optimal model comprehensively incorporated training set performance, internal validation, external validation, calibration, decision curve analysis, and model stability. This model showed no significant differences in accuracy and AUC across the training set, internal validation set, and external validation set. Priority was given to the accuracy of the external validation set.

### Intra- and inter-observer variability

The reliability of region of interest (ROI) delineation on CT images was assessed by evaluating the intra- and inter-observer variability using the Intraclass correlation coefficient (ICC). One researcher initially defined the ROI, while another researcher, who possessed over 10 years of experience in orthopedics, independently redefined the ROI for 30 randomly selected cases. The researchers were blinded to each other’s results throughout the process. ICC values were then calculated based on these 30 cases, and radiologic features showing ICCs higher than 0.9 were considered reliable in both the intra- and inter-observer categories. These features were subsequently chosen for further analysis.

### Radiomics feature extraction

The feature extraction process was performed utilizing the Pyradiomics Module (https://github.com/Radiomics/pyradiomics). To enhance the number of derived images, various filters, such as Laplacian of Gaussian filters and wavelets, were employed. All radiomics features were classified into seven categories, namely: (i) shape-based features; (ii) first-order features; (iii) gray-level dependence matrix (GLDM) features; (iv) gray-level size zone matrix (GLSZM) features; (v) neighboring gray-tone difference matrix (NGTDM) features; (vi) gray-level run-length matrix (GLRLM) features; and (vii) gray-level co-occurrence matrix (GLCM) features.

### Feature selection

In order to identify the most relevant features associated with the presence of osteoporosis, a meticulous feature selection process was implemented. Initially, the U test (p<0.05) was employed to identify features that exhibited significant differences between the osteoporosis and non-osteoporosis groups. Furthermore, to ensure the inclusion of only statistically significant and reliable features, those with intra-class correlation (ICC) coefficients lower than 0.9 were excluded from this step. This rigorous approach effectively reduced the number of features while maintaining their predictive power. To address the issue of multicollinearity, Pearson correlation analysis was conducted to examine the relationships between features. Calculation of correlation coefficients allowed for identification of feature pairs with values ≥0.9 or ≤-0.9. In such cases, only the feature demonstrating superior diagnostic performance was retained, thereby preventing redundancy within the model introduced by highly correlated features. Utilize the max relevance and min redundancy (mRMR) technique to eliminate redundant and irrelevant features, while retaining only 20 relevant features. To further refine the feature set, the least absolute shrinkage and selection operator (LASSO) logistic regression technique was employed. This technique allows for variable selection while penalizing coefficients, ultimately producing a more refined and robust feature set.

### Feature fusion

Using a pre-fusion approach, the clinical model and radiomics model were integrated separately to select the optimal Nomogram model for opportunistic osteoporosis screening based on radiomics techniques in the ROI region of hip joint CT combined with basic patient clinical information.

### Machine learning models

Seven common machine learning algorithms were used to train the extracted clinical features, radiomics features, and Nomogram model features. These algorithms include Logistic Regression (LR), Naive Bayes (NB), Support Vector Machine (SVM), Random Forest (RF), Extra Trees (ET), Gradient Boosting, and Multilayer Perceptron (MLP). The trained models were then utilized to detect osteoporosis in the test and validation datasets.

### 2D deep learning

Deep learning is a new research direction in the field of machine learning, bringing it closer to artificial intelligence. The method for recognizing and training two-dimensional images is known as two-dimensional deep learning. Before conducting two-dimensional deep learning, the largest cross-sectional area of the previously annotated region of interest (ROI) from the hip CT scan is delineated. This delineated largest cross-sectional area is then used as input data for the two-dimensional deep learning model. In this study, the two-dimensional deep learning models employed include AlexNet, VGG series, ResNet series, DenseNet series, Inception series, SqueezeNet series, ShuffleNetV2 series, MobileNet series, among others. All 2D deep learning models used in this study are presented in **Appendix Table 1**. During two-dimensional deep learning training, the batch size is set to 32, and stochastic gradient descent (SGD) is used as the optimizer for the deep learning training. Each model is trained for 100 epochs, with each epoch consisting of 1,800 iterations. The configuration parameters for deep learning training are as follows: ‘patch_size’: 64, ‘dim’: 1024, ‘depth’: 6, ‘heads’: 16, ‘mlp_dim’: 2048. The Grad-CAM visualization technique is used to generate recognition heatmaps. Grad-CAM is a technique for interpreting the decision-making process of deep learning models. It is a method for mapping the local importance of deep learning models, aiming to map the model’s decision-making process to specific regions of the input image.

### 3D deep learning

The method for recognizing and training three-dimensional images is known as three-dimensional deep learning. Before conducting three-dimensional deep learning, the entire on the hip CT scan is extracted individually. This extracted three-dimensional ROI is then used as input data for the deep learning model. In this study, the MedicalNet series, known for its effectiveness, is used as the three-dimensional deep learning model. MedicalNet is a pre-trained model specifically developed by Tencent for three-dimensional medical imaging. During the development of this model, Tencent aggregated multiple three-dimensional medical datasets into a large dataset and provided a complete set of pre-trained 3D-ResNet models along with corresponding learning and training code. All the code for three-dimensional image deep learning in MedicalNet has been open-sourced, available at https://www.oschina.net/p/medicalnet?hmsr=aladdin1e1. The models used in this study include various 3D-ResNet series such as 3DShuffleNet, 3DResNet10, 3DResNet18, 3DResNet34, 3DResNet50, 3DResNet152, and 3DResNet200. During the three-dimensional deep learning training, the batch size was set to 32, and stochastic gradient descent (SGD) was used as the optimizer for deep learning training. Each model underwent 100 epochs, with each epoch comprising 1800 iterations. The best-performing model was selected for further comparison.

### Statistical analysis

The baseline data of the patients were analyzed using Python (pandas and numpy). The continuous variables were reported as mean ± standard deviation, while categorical variables were described using frequencies and percentages. The distribution of continuous variables was assessed using the Kolmogorov-Smirnov test, and the homogeneity of continuous variances was evaluated using the Levene test. Inter-group differences were compared using the Student’s t-test, depending on the variable distribution, while categorical variables were analyzed using either the Chi-squared test or Fisher’s exact test. Statistical significance was defined as a p-value < 0.05.The performance of predictive models was evaluated using the Area Under the Curve (AUC), and the 95% confidence interval (CI) of the AUC was calculated using the bootstrap method with 1000 intervals. To compare the AUCs of different models, the DeLong testing method was employed, allowing for a statistical assessment of the differences in performance metrics between the models.

## Results

### Screening and efficacy of clinical models

**Figure 2:**
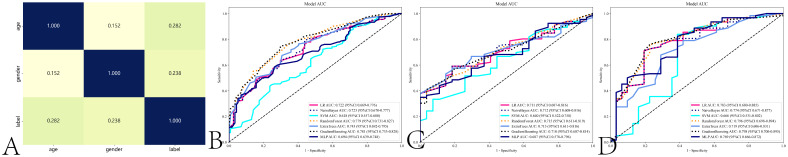
The process and efficacy of establishing a clinical model. **(A)** Results of Spearman correlation analysis between gender, age, and osteoporosis. **(B)** Application of clinical models to identify the AUC for opportunistic osteoporosis in the training group. **(C)** Application of clinical models to identify the AUC for opportunistic osteoporosis in the internal testing group. **(D)** Utilization of Clinical Models to Assess the AUC for Opportunistic Osteoporosis in the External Validation Group.

Patients were screened based on gender and age. In both univariate and multivariate regression analyses, the P-values for these two indicators were less than 0.01, indicating that both factors are associated with the occurrence of osteoporosis. The results of the univariate and multivariate analyses are presented in **Table 2**. Based on the results of these analyses, this study proposes to use gender and age to establish a clinical model for opportunistic osteoporosis screening. Spearman correlation analysis calculated the correlation coefficient between gender and osteoporosis as 0.238, and the correlation coefficient between age and osteoporosis as 0.282. The results of the Spearman correlation analysis are shown in **Figure 2A**. The efficacy of the clinical model established based on gender and age for screening opportunistic osteoporosis is presented in **Appendix Table 2** and **Figures 2B-D**.

**Table 2:** Process of screening clinical factors using univariate analysis and multivariate analysis.

Single factor analysis	Log(OR)	Lower 95%CI	Upper 95%CI	OR	OR lower 95%CI	OR upper 95%CI	*P* value
Gender	0.227	0.157	0.297	1.254	1.170	1.346	<0.01
Age	0.010	0.007	0.012	1.010	1.007	1.012	<0.01
Multi factor analysis	Log(OR)	Lower 95%CI	Upper 95%CI	OR	OR lower 95%CI	OR upper 95%CI	*P* value
Age	0.009	0.006	0.011	1.009	1.006	1.011	<0.01
Gender	0.185	0.116	0.254	1.203	1.123	1.289	<0.01

### Screening and efficacy of radiomic models

**Figure 3:**
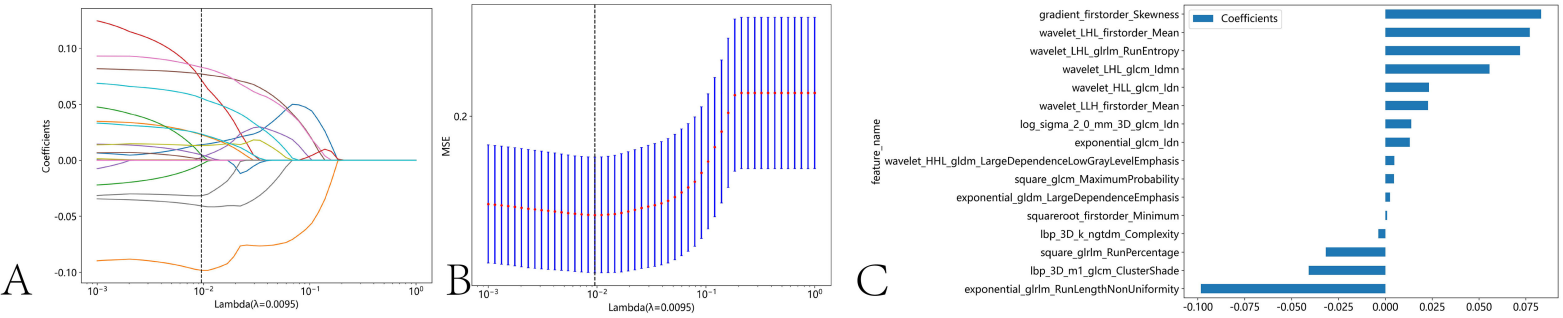
The screening process of radiomics models. **(A, B)**, the process of performing lasso regression; **(C)**, the selected radiomics features.

After screening, 15 radiomics features were selected for the establishment of radiomics models. The screening process and the resulting radiomics model are presented in **Appendix Table 3** and **Figure 3**. The efficacy of radiomics model screening for opportunistic osteoporosis is shown in **Appendix Table 4**.

### Effectiveness of the nomogram model

**Figure 4:**
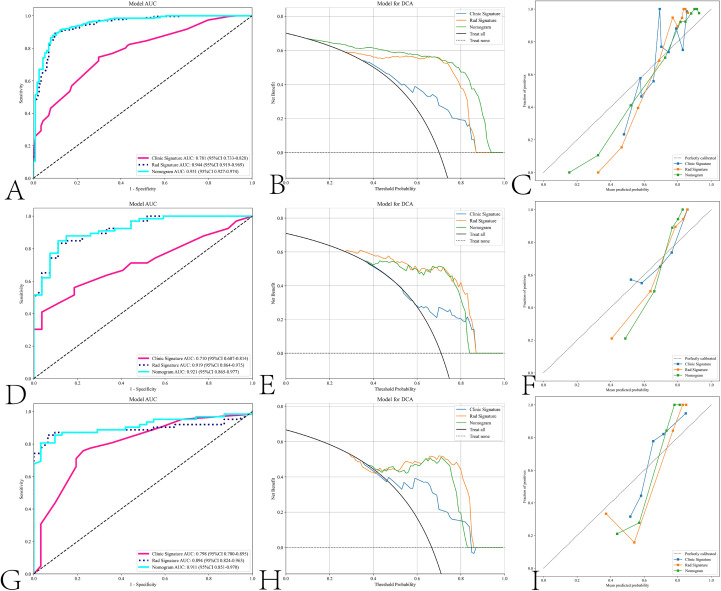
AUC curves, DCA curves, and DCA calibration curves of the most effective nomogram model for screening opportunistic osteoporosis. **(A)** AUC curve for screening opportunistic osteoporosis using the GradientBoosting algorithm combined with the nomogram model in the training group; **(B)** DCA curve for screening opportunistic osteoporosis using the GradientBoosting algorithm combined with the nomogram model in the training group; **(C)** DCA calibration curve for screening opportunistic osteoporosis using the GradientBoosting algorithm combined with the nomogram model in the training group; **(D)** AUC curve for screening opportunistic osteoporosis using the GradientBoosting algorithm combined with the nomogram model in the test group; **(E)** DCA curve for screening opportunistic osteoporosis using the GradientBoosting algorithm combined with the nomogram model in the test group; **(F)** DCA calibration curve for screening opportunistic osteoporosis using the GradientBoosting algorithm combined with the Nomogram model in the test group; **(G)** AUC curve for screening opportunistic osteoporosis using the GradientBoosting algorithm combined with the nomogram model in the validation group; **(H)** DCA curve for screening opportunistic osteoporosis using the GradientBoosting algorithm combined with the nomogram model in the validation group; **(I)** DCA calibration curve for screening opportunistic osteoporosis using the GradientBoosting algorithm combined with the nomogram model in the validation group.

Integrate radiomics models with clinical models to develop a Nomogram model. Among all Nomogram models, the GradientBoosting machine learning algorithm demonstrated the best performance in both the test and validation groups. The performance of the Nomogram model in screening opportunistic osteoporosis is presented in **Appendix Table 5**. The efficacy of utilizing this algorithm in conjunction with the Nomogram model for opportunistic osteoporosis screening is illustrated in **Figure 4**.

### Effectiveness of 2D deep learning models

**Figure 5:**
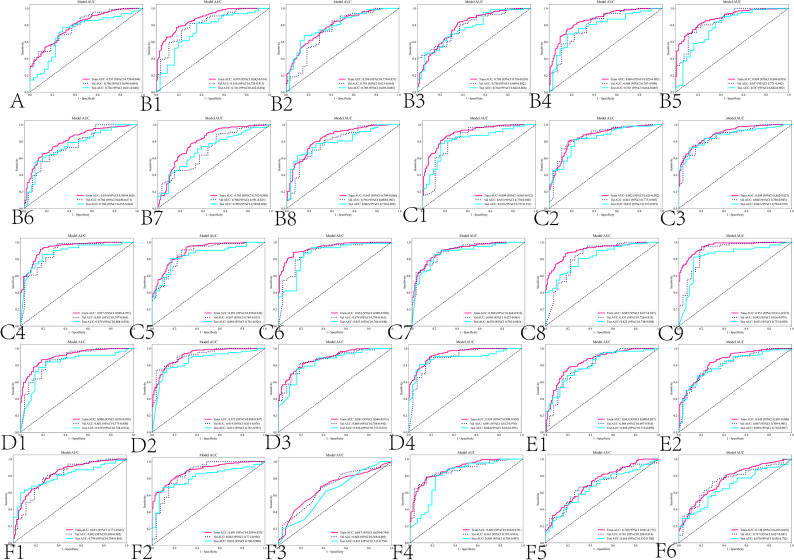
Performance of 2D deep learning models. **(A)** Performance of the AlexNet model. **(B1)** Performance of the VGG11 model; **(B2)** Performance of the VGG11_BN model; **(B#)** Performance of the VGG13 model; **(B4)** Performance of the VGG13_BN model; **(B5)** Performance of the VGG16 model; **(B6)** Performance of the VGG16_BN model; **(B7)** Performance of the VGG19 model; **(B8)** Performance of the VGG19_BN model. **(C1)** Performance of the ResNet18 model; **(C2)** Performance of the ResNet34 model; **(C3)** Performance of the ResNet50 model; **(C4)** Performance of the ResNet101 model; **(C5)** Performance of the ResNet152 model; **(C6)** Performance of the ResNeXt50_32x4d model; **(C7)** Performance of the ResNeXt101_32x8d model; **(C8)** Performance of the Wide ResNet50_2 model; **(C9)** Performance of the Wide ResNet101_2 model. **(D1)** Performance of the DenseNet121 model; **(D2)** Performance of the DenseNet161 model; **(D3)** Performance of the DenseNet169 model; **(D4)** Performance of the DenseNet201 model. **(E1)** Performance of the GoogleNet model; **(E2)** Performance of the Inception_v3 model. **(F1)** Performance of the SqueezeNet1.0 model; **(F2)** Performance of the SqueezeNet1.1 model; **(F3)** Performance of the ShuffleNet V2 x1.0 model; **(F4)** Performance of the MobileNet V2 model; **(F5)** Performance of the MobileNet V3 Large model; **(F6)** Performance of the MobileNet V3 Small model.

**Figure 6:**
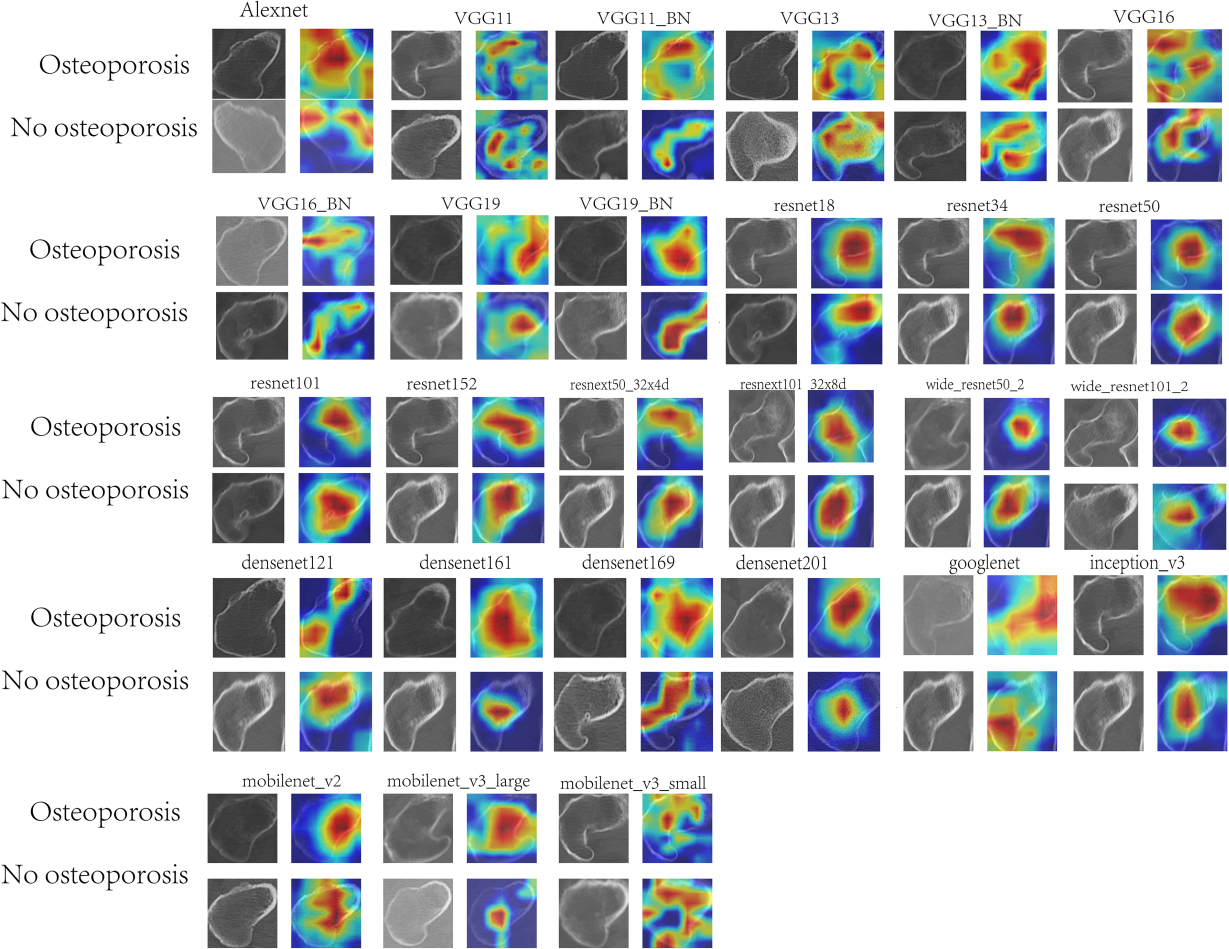
Heat map of 2D deep learning model.

The efficacy of using a 2D deep learning model combined with the maximum cross-sectional area of the patient’s hip joint CT ROI to identify the presence of osteoporosis is demonstrated in **Figure 5** and **Appendix Table 6**. **Figure 6** shows the heatmap generated by Grad-CAM for AlexNet recognition. Among the three-dimensional deep learning models, the three-dimensional ResNet34 exhibited the best performance in the validation group, with an accuracy of 0.806 and an AUC of 0.889 for screening opportunistic osteoporosis.

### Effectiveness of 3D deep learning models

**Figure 7:**
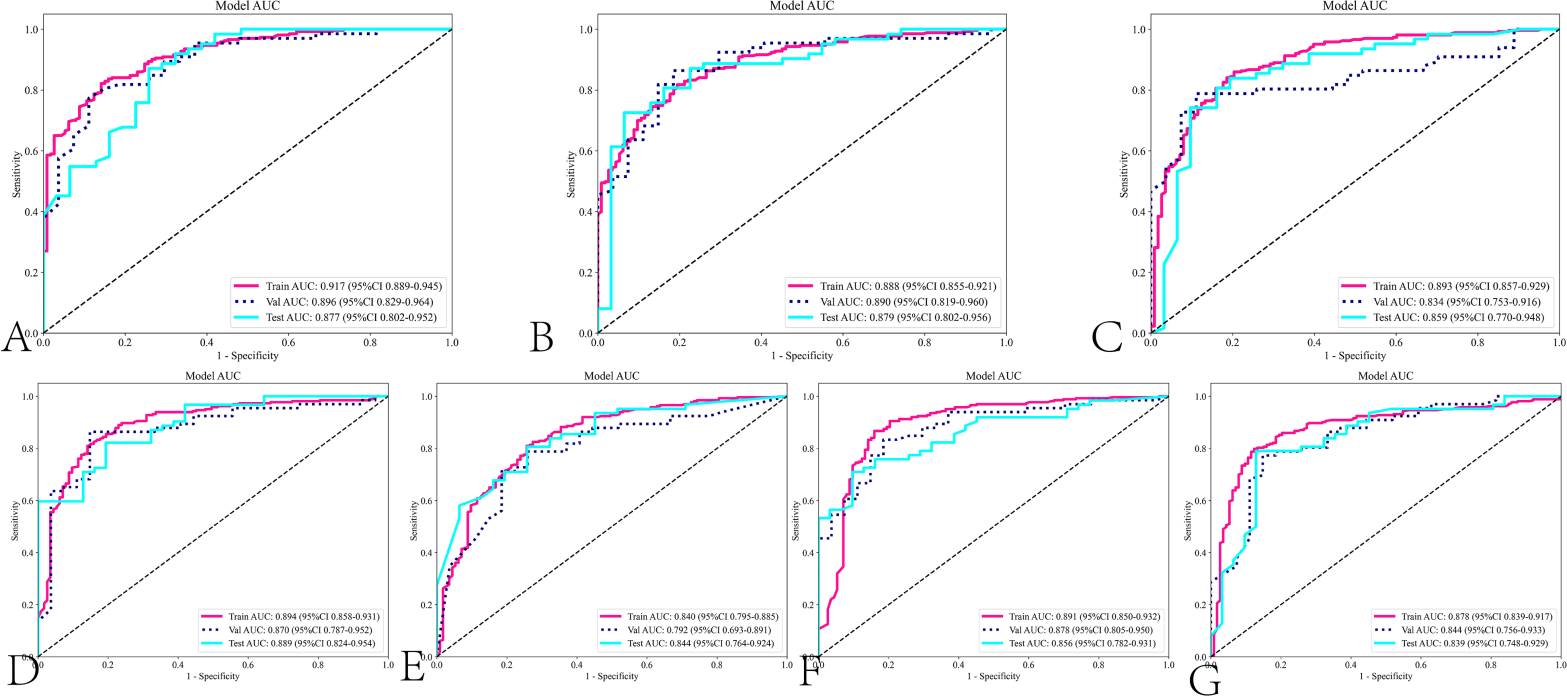
Efficiency of screening opportunistic osteoporosis based on three-dimensional deep learning models trained on the ROI region of hip joint CT: **(A)** Efficiency of 3D ShuffleNet; **(B)** Efficiency of 3D resnet10; **(C)** Efficiency of 3D resnet18; **(D)** Efficiency of 3D resnet34; **(E)** Efficiency of 3D resnet50; **(F)** Efficiency of 3D resnet152; **(G)** Efficiency of 3D resnet200.

The efficacy of screening opportunistic osteoporosis using three-dimensional deep learning after training on the ROI area of patient hip joint CT is demonstrated in **Appendix Table 7** and **Figure 7**. Among the three-dimensional deep learning models, the 3D ResNet34 exhibited the best performance in the validation group, with an accuracy of 0.806 and an AUC of 0.889 for screening opportunistic osteoporosis in the validation group.

### Comparison of the efficacy of nomogram model, radiomics model, and clinical model

The comparison of AUCs between the Nomogram model, radiomics model, and clinical model is demonstrated in **Appendix Table 8**. In all machine learning algorithms across training groups, internal testing groups, and external validation groups, the AUC of the Nomogram model was superior to that of the clinical model, with statistically significant differences.

### Comparison of model efficiency

The comparison of AUCs between the best Nomogram model, best three-dimensional deep learning model, and best two-dimensional deep learning model for detecting opportunistic osteoporosis using DeLong tests is presented in **Table 3**. Among all Nomogram models (radiomics model + clinical model), the GradientBoosting machine learning algorithm exhibited the best performance in the validation group, with an accuracy of 0.849 and an AUC of 0.911 for screening opportunistic osteoporosis. Among all two-dimensional deep learning models, DenseNet201 demonstrated the best performance in the validation group, with an accuracy of 0.817 and an AUC of 0.884. In lightweight two-dimensional deep learning models, SqueezeNet1_1 showed the best performance in the validation group, with an accuracy of 0.753 and an AUC of 0.810. Among three-dimensional deep learning models, the three-dimensional ResNet34 had the best performance in the validation group, with an accuracy of 0.806 and an AUC of 0.889. The efficacy comparison of the best Nomogram model, best three-dimensional deep learning model, and best two-dimensional deep learning model is shown in **Table 3**.

**Table 3:** The performance of the best nomogram model, best 2d deep learning model, best 3D deep learning model, and best lightweight 2D deep learning model.

Group	Nomogram model vs 2D deep learning model	Nomogram model vs 3D deep learning model	2D deep learning model vs 3D deep learning model
Training group	0.34	<0.05	<0.05
Internal testing group	0.41	0.09	0.58
External Verification Group	0.49	0.56	0.91

## Discussion

Our study presents initial evidence supporting the utility of radiomics and deep learning techniques for the identification of osteoporosis in patients with hip injuries. Specifically, we utilized CT scans of unaffected hip joints to perform this analysis. The application of these techniques extends to patients who undergo hip CT scans due to hip joint pain, as it enables the preliminary determination of osteoporosis presence in such cases.

In this study, patient clinical information was initially utilized to establish a clinical model for determining the presence of osteoporosis. Following univariate and multivariate analysis, it was found that gender and age were correlated factors associated with osteoporosis occurrence. As individuals age, the bone formation process decreases while bone loss increases, rendering the elderly population more susceptible to osteoporosis (20). Postmenopausal women are particularly prone to osteoporosis due to hormonal changes that occur after menopause, such as a decline in estrogen, growth hormone, and thyroid hormone levels, as well as an increase in parathyroid hormone levels (21). These altered hormone levels, whether individually or in combination, contribute to a reduction in osteoblast activity and an increase in osteoclast activity, leading to accelerated bone turnover and increased bone loss (22). Despite the confirmed correlation between age and gender with the occurrence of osteoporosis, the clinical model constructed using these factors alone does not yield satisfactory results in determining the presence of osteoporosis among patients. In the test group, the best accuracy of this model in identifying osteoporosis was found to be only 0.753, with an area under the curve (AUC) of 0.796. Compared to clinical models, radiomics models have greatly enhanced their effectiveness in diagnosing osteoporosis in patients. Radiomics involves the extraction of high-dimensional data from radiological images, which has previously been employed in oncology to enhance diagnostic and prognostic capabilities, with the goal of advancing precision medicine (23). Numerous scholars have employed radiomics techniques to discern pertinent attributes of the lumbar spine in order to ascertain the presence of osteoporosis in patients, yielding commendable outcomes (24, 25). In our investigation, this model exhibited remarkable efficacy in the diagnosis of osteoporosis. In the test group, the accuracy of radiomic model in identifying osteoporosis was found to be 0.882, with an area under the curve (AUC) of 0.926. However, not all machine learning algorithms outperform clinical models in terms of efficacy for radiomics models. To further enhance the efficacy of screening opportunistic osteoporosis using hip joint CT combined with radiomics technology, the authors adopted a pre-fusion approach, integrating the radiomics model with the clinical model to form a Nomogram model. The efficacy of this model surpasses that of clinical models across all machine learning algorithms and is statistically significant.

Deep learning technology is extensively utilized for the diagnosis of osteoporosis in patients. A recent study showcased the employment of the DCNN model for discerning the presence of osteoporosis on lumbar X-ray scans, yielding promising outcomes (26). In our investigation, we employed 30 kinds of 2D Deep Learning Models to train hip CT images and successfully identified the existence of osteoporosis, attaining equally remarkable results (27). Moreover, our research exhibits proficiency in recognizing not only 2D images but also 3D images. In the test group, the best accuracy of 2D deep learning model in identifying osteoporosis was found to be 0.817, with an AUC of 0.884. In the test group, the best accuracy of 3D deep learning model in identifying osteoporosis was found to be 0.806, with an AUC of 0.889. This aspect further substantiates the viability of utilizing hip CT images as a means to assess the presence of osteoporosis, regardless of employing 2D deep learning to extract the maximum cross-sectional area of the RO or engaging in 3D deep learning analysis encompassing the entirety of the ROI. Furthermore, visualization technology plays a crucial role in conducting in-depth analysis of hip CT images. It has been observed that the visualization area predominantly lies between the femoral neck and trochanter in most patients. Studies have shown that femoral neck fractures and intertrochanteric fractures are the most frequently occurring osteoporotic fractures (28). Consequently, it is likely that deep learning algorithms prioritize these regions for identification purposes.

The osteoporotic changes in bone microarchitecture manifest as relatively global textural alterations on CT images rather than local fine features. The traditional texture and wavelet features extracted by radiomics can effectively capture such changes, thereby narrowing the performance gap with deep learning to some extent. The sample size of this study may still be relatively small for training deep neural networks with a large number of parameters, whereas radiomics combined with LASSO regression has better adaptability to small datasets and a lower risk of overfitting. This also explains why, although deep learning performed well on the training set, its advantage was less evident in the external validation set. Furthermore, there was no significant difference in performance between 2D and 3D deep learning, suggesting that 2D analysis using the maximum cross-sectional area of the ROI already captures most of the osteoporosis-relevant imaging information. 3D convolutional neural networks have a larger number of parameters and are more prone to overfitting with limited sample sizes, which may be the main reason they failed to demonstrate a significant advantage.

In terms of performance, the Nomogram model achieved an AUC of 0.911 in the external validation set, significantly outperforming the clinical model alone. This improvement has clear clinical significance, as it can reduce unnecessary DXA referrals. Therefore, its increased model complexity is justified in large medical institutions. However, the clinical deployment of complex models also faces practical challenges: 3D deep learning requires GPU acceleration and has longer inference times; the “black box” nature of deep learning models may affect clinicians’ trust; and models require regular updates and recalibration to maintain robustness across different devices.

Our research does have certain limitations. Firstly, future multicenter studies are necessary to validate the experimental findings. Additionally, the delineation of ROI areas was performed manually, and further exploration of automatic delineation methods is warranted in future research.

## Conclusion

Our study suggests that the Nomogram model, which combines radiomics techniques based on ROI regions on hip joint CT with gender and age integration, can be considered for opportunistic osteoporosis screening. This is true across various machine learning algorithms, as well as two-dimensional deep learning models like DenseNet201 and three-dimensional deep learning models like ResNet34. However, we emphasize that prospective validation and calibration are required before any clinical implementation.

## Data Availability

The raw data supporting the conclusions of this article will be made available by the authors, without undue reservation.
