# Supplementary material for: Screening opportunistic osteoporosis through multimodal techniques of hip joint CT images: exploring 2D and 3D deep learning, radiomics, clinical data, and their integration
Source: Front Endocrinol (Lausanne). 2026 Jul 17;17:1838830. doi: 10.3389/fendo.2026.1838830 (PMC13423716; doi:10.3389/fendo.2026.1838830)
Supplement: Supplementary file 1 [file DataSheet1.pdf]

Appendix Table1. The 2D deep learning model used in this study.

Model Series	Model Name
AlexNet	alexnet
VGG	vgg11, vgg11_bn, vgg13, vgg13_bn, vgg16, vgg16_bn, vgg19_bn, vgg19
ResNet	resnet18, resnet34, resnet50, resnet101, resnet152, resnext50_32x4d, resnext101_32x8d, wide_resnet50_2, wide_resnet101_2
DenseNet	densenet121, densenet169, densenet201, densenet161
Inception	googlenet, inception_v3
Lightweight network	squeezenet1_0, squeezenet1_1, shufflenet_v2_x1_0,mobilenet_v2, mobilenet_v3_large, mobilenet_v3_small

Appendix Table2. Efficacy of Clinical Models

Group	Model name	Accuracy	AUC	95% CI	Sensitivity	Specificity
Training group	LR	0.656	0.722	0.669 - 0.776	0.637	0.702
Internal testing group	LR	0.656	0.711	0.607 - 0.816	0.591	0.815
External Verification Group	LR	0.720	0.783	0.680 - 0.885	0.677	0.806
Training group	NaiveBayes	0.722	0.723	0.670 - 0.777	0.790	0.561
Internal testing group	NaiveBayes	0.656	0.712	0.608 - 0.816	0.591	0.815
External Verification Group	NaiveBayes	0.720	0.774	0.671 - 0.877	0.677	0.806
Training group	SVM	0.538	0.618	0.557 - 0.680	0.446	0.754
Internal testing group	SVM	0.505	0.640	0.522 - 0.758	0.333	0.926
External Verification Group	SVM	0.742	0.666	0.531 - 0.802	0.806	0.613
Training group	RandomForest	0.732	0.779	0.731 - 0.827	0.768	0.649
Internal testing group	RandomForest	0.559	0.715	0.611 - 0.819	0.394	0.963
External Verification Group	RandomForest	0.753	0.796	0.698 - 0.894	0.790	0.677
Training group	ExtraTrees	0.680	0.743	0.692 - 0.795	0.674	0.693
Internal testing	ExtraTrees	0.645	0.713	0.611 -	0.576	0.815

group				0.816		
External Verification Group	ExtraTrees	0.667	0.719	0.606 - 0.831	0.645	0.667
Training group	GradientBoosting	0.732	0.781	0.733 - 0.828	0.745	0.702
Internal testing group	GradientBoosting	0.634	0.710	0.607 - 0.814	0.561	0.815
External Verification Group	GradientBoosting	0.742	0.798	0.700 - 0.895	0.710	0.806
Training group	MLP	0.601	0.694	0.639 - 0.748	0.539	0.746
Internal testing group	MLP	0.538	0.687	0.578 - 0.796	0.348	1.000
External Verification Group	MLP	0.742	0.769	0.666 - 0.872	0.806	0.613

Appendix Table3. Selected radiomic features

Image type	Feature type	Feature name
log_sigma_2_0_mm_3D	glcm	Idn
exponential	glrlm	RunLengthNonUniformity
lbp_3D_k	ngtdm	Complexity
wavelet_LHL	glrlm	RunEntropy
exponential	gldm	LargeDependenceEmphasis
wavelet_LHL	firstorder	Mean
gradient	firstorder	Skewness
lbp_3D_m1	glcm	ClusterShade
wavelet_LHL	glcm	Idmn
wavelet_LLH	firstorder	Mean
square	glcm	MaximumProbability
wavelet_HHL	gldm	LargeDependenceLowGrayLevelEmphasis
squareroot	firstorder	Minimum
square	glrlm_	RunPercentage
exponential	glcm	Idn

Appendix Table4. Efficacy of Radiomics Models

Group	Model name	Accuracy	AUC	95% CI	Sensitivity	Specificity
Training group	LR	0.782	0.890	0.857 - 0.924	0.738	0.886
Internal testing group	LR	0.828	0.879	0.803 - 0.955	0.833	0.815
External Verification Group	LR	0.871	0.881	0.810 - 0.952	0.823	0.968
Training group	NaiveBayes	0.790	0.856	0.815 - 0.897	0.779	0.823
Internal testing group	NaiveBayes	0.785	0.841	0.741 - 0.940	0.742	0.923
External Verification Group	NaiveBayes	0.871	0.874	0.791 - 0.958	0.823	0.968
Training group	SVM	0.858	0.938	0.913 - 0.963	0.831	0.921
Internal testing group	SVM	0.839	0.891	0.809 - 0.974	0.803	0.926
External Verification Group	SVM	0.860	0.893	0.826 - 0.960	0.806	0.968
Training group	RandomForest	0.824	0.921	0.891 - 0.951	0.790	0.904
Internal testing group	RandomForest	0.839	0.888	0.811 - 0.964	0.803	0.926
External Verification Group	RandomForest	0.871	0.923	0.868 - 0.978	0.823	0.968
Training group	ExtraTrees	0.795	0.874	0.837 - 0.911	0.787	0.816
Internal testing group	ExtraTrees	0.817	0.889	0.814 - 0.963	0.803	0.852

External Verification Group	ExtraTrees	0.882	0.926	0.872 - 0.980	0.855	0.935
Training group	GradientBoosting	0.895	0.944	0.919 - 0.969	0.891	0.904
Internal testing group	GradientBoosting	0.849	0.919	0.864 - 0.975	0.833	0.889
External Verification Group	GradientBoosting	0.871	0.894	0.824 - 0.963	0.839	0.935
Training group	MLP	0.814	0.887	0.853 - 0.921	0.801	0.842
Internal testing group	MLP	0.785	0.877	0.802 - 0.952	0.727	0.926
External Verification Group	MLP	0.882	0.902	0.839 - 0.965	0.823	1.000

Appendix Table 5. Efficacy of Nomogram Models

Group	Model name	Accuracy	AUC	95% CI	Sensitivity	Specificity
Training group	LR	0.798	0.893	0.860 - 0.927	0.768	0.868
Internal testing group	LR	0.828	0.877	0.800 - 0.953	0.833	0.815
External Verification Group	LR	0.871	0.899	0.835 - 0.962	0.806	1.000
Training group	NaiveBayes	0.790	0.869	0.830 - 0.909	0.768	0.842
Internal testing group	NaiveBayes	0.774	0.857	0.776 - 0.937	0.727	0.889
External Verification Group	NaiveBayes	0.882	0.926	0.873 - 0.979	0.855	0.935
Training group	SVM	0.856	0.937	0.913 - 0.962	0.828	0.921
Internal testing group	SVM	0.828	0.891	0.809 - 0.974	0.788	0.926
External Verification Group	SVM	0.860	0.893	0.826 - 0.960	0.806	0.968
Training group	Random Forest	0.877	0.930	0.903 - 0.957	0.903	0.816
Internal testing group	Random Forest	0.839	0.907	0.845 - 0.970	0.833	0.852
External Verification Group	Random Forest	0.828	0.922	0.870 - 0.975	0.774	0.935
Training group	ExtraTrees	0.808	0.896	0.863 - 0.929	0.779	0.877

Internal testing group	ExtraTrees	0.828	0.896	0.833 - 0.960	0.833	0.815
External Verification Group	ExtraTrees	0.828	0.919	0.864 - 0.974	0.774	0.935
Training group	Gradient Boosting	0.887	0.951	0.927 - 0.974	0.876	0.912
Internal testing group	Gradient Boosting	0.849	0.921	0.865 - 0.977	0.833	0.889
External Verification Group	Gradient Boosting	0.849	0.911	0.851 - 0.970	0.790	0.968
Training group	MLP	0.785	0.888	0.854- 0.922	0.734	0.904
Internal testing group	MLP	0.774	0.875	0.799 - 0.951	0.712	0.926
External Verification Group	MLP	0.882	0.904	0.843 - 0.966	0.823	1.000

Appendix Table6. Efficacy of 2D deep learning models

Group	Model name	Accuracy	AUC	95% CI	Sensitivity	Specificity
Training group	Alexnet	0.740	0.797	0.750-0.844	0.779	0.649
Internal testing group	Alexnet	0.785	0.796	0.698-0.894	0.879	0.556
External Verification Group	Alexnet	0.742	0.726	0.611-0.840	0.758	0.710
Training group	vgg11	0.793	0.878	0.842-0.914	0.787	0.807
Internal testing group	vgg11	0.774	0.818	0.724-0.913	0.803	0.704
External Verification Group	vgg11	0.753	0.743	0.632-0.854	0.806	0.645
Training group	vgg11_bn	0.764	0.788	0.739-0.837	0.843	0.579
Internal testing group	vgg11_bn	0.763	0.743	0.621-0.864	0.833	0.593
External Verification Group	vgg11_bn	0.710	0.789	0.689-0.888	0.645	0.839
Training group	vgg13	0.774	0.788	0.738-0.839	0.831	0.64
Internal testing group	vgg13	0.742	0.786	0.689-0.882	0.788	0.63
External Verification Group	vgg13	0.677	0.764	0.662-0.866	0.661	0.71
Training group	vgg13_bn	0.774	0.864	0.825-0.903	0.745	0.842
Internal testing group	vgg13_bn	0.785	0.808	0.707-0.909	0.848	0.630
External Verification Group	vgg13_bn	0.731	0.785	0.684-0.885	0.677	0.839
Training group	vgg16	0.801	0.884	0.849-0.918	0.798	0.807
Internal testing group	vgg16	0.774	0.857	0.771-0.942	0.758	0.815

External Verification Group	vgg16	0.763	0.787	0.682-0.892	0.823	0.645
Training group	vgg16_bn	0.701	0.814	0.768-0.860	0.640	0.842
Internal testing group	vgg16_bn	0.667	0.760	0.649-0.871	0.621	0.778
External Verification Group	vgg16_bn	0.677	0.760	0.655-0.864	0.597	0.839
Training group	vgg19	0.738	0.795	0.742-0.848	0.753	0.702
Internal testing group	vgg19	0.742	0.708	0.591-0.825	0.848	0.481
External Verification Group	vgg19	0.677	0.709	0.599-0.820	0.694	0.645
Training group	vgg19_bn	0.766	0.843	0.799-0.886	0.757	0.789
Internal testing group	vgg19_bn	0.763	0.796	0.690-0.902	0.803	0.667
External Verification Group	vgg19_bn	0.710	0.802	0.710-0.894	0.613	0.903
Training group	resnet18	0.840	0.899	0.865-0.932	0.861	0.789
Internal testing group	resnet18	0.806	0.835	0.730-0.940	0.818	0.778
External Verification Group	resnet18	0.785	0.819	0.727-0.911	0.758	0.839
Training group	resnet34	0.811	0.862	0.821-0.902	0.801	0.833
Internal testing group	resnet34	0.796	0.861	0.773-0.949	0.788	0.815
External Verification Group	resnet34	0.796	0.833	0.747-0.919	0.806	0.774
Training group	resnet50	0.782	0.894	0.862-0.927	0.742	0.877
Internal testing group	resnet50	0.774	0.868	0.790-0.945	0.742	0.852
External	resnet50	0.742	0.867	0.794-0.939	0.661	0.903

Verification Group						
Training group	resnet101	0.885	0.927	0.898-0.955	0.91	0.825
Internal testing group	resnet101	0.882	0.881	0.797-0.965	0.970	0.667
External Verification Group	resnet101	0.817	0.879	0.804-0.954	0.823	0.806
Training group	resnet152	0.869	0.893	0.859-0.928	0.940	0.702
Internal testing group	resnet152	0.849	0.867	0.784-0.951	0.894	0.741
External Verification Group	resnet152	0.806	0.844	0.761-0.926	0.790	0.839
Training group	resnext50_32x4d	0.874	0.934	0.909-0.958	0.876	0.868
Internal testing group	resnext50_32x4d	0.839	0.878	0.794-0.961	0.864	0.778
External Verification Group	resnext50_32x4d	0.828	0.853	0.766-0.940	0.855	0.774
Training group	resnext101_32x8d	0.856	0.900	0.868-0.932	0.906	0.737
Internal testing group	resnext101_32x8d	0.839	0.894	0.823-0.965	0.864	0.778
External Verification Group	resnext101_32x8d	0.828	0.870	0.792-0.948	0.839	0.806
Training group	wide_resnet50_2	0.850	0.907	0.877-0.937	0.861	0.825
Internal testing group	wide_resnet50_2	0.839	0.831	0.726-0.935	0.909	0.667
External Verification Group	wide_resnet50_2	0.763	0.823	0.738-0.908	0.790	0.710
Training group	wide_resnet101_2	0.895	0.951	0.931-0.972	0.936	0.798
Internal testing group	wide_resnet101_2	0.892	0.892	0.810-0.974	0.939	0.778

External Verification Group	wide_resnet101_2	0.828	0.831	0.733-0.929	0.806	0.871
Training group	densenet121	0.848	0.908	0.878-0.938	0.861	0.816
Internal testing group	densenet121	0.817	0.863	0.775-0.950	0.833	0.778
External Verification Group	densenet121	0.785	0.819	0.724-0.914	0.758	0.839
Training group	densenet161	0.822	0.917	0.888-0.947	0.805	0.860
Internal testing group	densenet161	0.796	0.914	0.851-0.976	0.727	0.963
External Verification Group	densenet161	0.785	0.851	0.767-0.935	0.742	0.871
Training group	densenet169	0.808	0.881	0.846-0.915	0.816	0.789
Internal testing group	densenet169	0.806	0.864	0.786-0.942	0.803	0.815
External Verification Group	densenet169	0.774	0.836	0.751-0.921	0.742	0.839
Training group	densenet201	0.874	0.934	0.909-0.959	0.899	0.816
Internal testing group	densenet201	0.860	0.891	0.812-0.970	0.879	0.815
External Verification Group	densenet201	0.817	0.884	0.814-0.955	0.774	0.903
Training group	googlenet	0.780	0.843	0.800-0.887	0.775	0.789
Internal testing group	googlenet	0.720	0.804	0.697-0.910	0.697	0.778
External Verification Group	googlenet	0.710	0.806	0.712-0.899	0.661	0.806
Training group	inception_v3	0.801	0.843	0.801-0.886	0.831	0.728
Internal testing group	inception_v3	0.742	0.807	0.709-0.905	0.727	0.778
External Verification Group	inception_v3	0.710	0.803	0.710-0.897	0.645	0.839

Training group	squeezenet1_0	0.769	0.819	0.773-0.865	0.809	0.675
Internal testing group	squeezenet1_0	0.753	0.802	0.699-0.905	0.758	0.741
External Verification Group	squeezenet1_0	0.699	0.794	0.704-0.885	0.581	0.935
Training group	squeezenet1_1	0.814	0.891	0.859-0.923	0.824	0.789
Internal testing group	squeezenet1_1	0.796	0.863	0.771-0.956	0.803	0.778
External Verification Group	squeezenet1_1	0.753	0.810	0.720-0.9000	0.677	0.903
Training group	shufflenet_v2_x1_0	0.654	0.687	0.630-0.744	0.663	0.632
Internal testing group	shufflenet_v2_x1_0	0.570	0.685	0.568-0.803	0.515	0.704
External Verification Group	shufflenet_v2_x1_0	0.505	0.633	0.512-0.754	0.403	0.710
Training group	mobilenet_v2	0.798	0.884	0.850-0.919	0.775	0.851
Internal testing group	mobilenet_v2	0.796	0.867	0.791-0.943	0.773	0.852
External Verification Group	mobilenet_v2	0.785	0.805	0.703-0.907	0.790	0.774
Training group	mobilenet_v3_large	0.667	0.720	0.665-0.775	0.667	0.667
Internal testing group	mobilenet_v3_large	0.667	0.701	0.588-0.814	0.652	0.704
External Verification Group	mobilenet_v3_large	0.613	0.666	0.552-0.780	0.565	0.710

Training group	mobilenet_v3_small	0.738	0.748	0.695-0.801	0.801	0.588
Internal testing group	mobilenet_v3_small	0.710	0.737	0.627-0.847	0.712	0.704
External Verification Group	mobilenet_v3_small	0.624	0.670	0.558-0.782	0.597	0.677

Appendix Table7. Efficacy of 3D deep learning models

Group	Model name	Accuracy	AUC	95% CI	Sensitivity	Specificity
Training group	3DShuffleNet	0.824	0.917	0.889-0.945	0.809	0.860
Internal testing group	3DShuffleNet	0.785	0.896	0.829-0.964	0.742	0.889
External Verification Group	3DShuffleNet	0.817	0.877	0.802-0.952	0.855	0.742
Training group	3Dresnet10	0.811	0.888	0.855-0.921	0.813	0.807
Internal testing group	3Dresnet10	0.839	0.890	0.819-0.960	0.848	0.815
External Verification Group	3Dresnet10	0.785	0.879	0.802-0.956	0.710	0.935
Training group	3Dresnet18	0.837	0.893	0.857-0.929	0.854	0.798
Internal testing group	3Dresnet18	0.806	0.834	0.753-0.916	0.773	0.889
External Verification Group	3Dresnet18	0.817	0.859	0.770-0.948	0.823	0.806
Training group	3Dresnet34	0.822	0.894	0.858-0.931	0.805	0.860
Internal testing group	3Dresnet34	0.849	0.870	0.787-0.952	0.848	0.852
External Verification Group	3Dresnet34	0.806	0.889	0.82-0.954	0.806	0.806
Training group	3Dresnet50	0.787	0.840	0.795-0.885	0.805	0.746
Internal testing group	3Dresnet50	0.763	0.792	0.693-0.891	0.773	0.741
External Verification	3Dresnet50	0.763	0.844	0.764-0.924	0.774	0.742

Group						
Training group	3Dresnet152	0.856	0.891	0.850-0.932	0.861	0.842
Internal testing group	3Dresnet152	0.817	0.878	0.805-0.950	0.818	0.815
External Verification Group	3Dresnet152	0.763	0.856	0.782-0.931	0.694	0.903
Training group	3Dresnet200	0.816	0.878	0.839-0.917	0.790	0.877
Internal testing group	3Dresnet200	0.785	0.844	0.756-0.933	0.758	0.852
External Verification Group	3Dresnet200	0.806	0.839	0.748-0.929	0.774	0.871

Appendix Table 8. Comparison of the Efficacy of Nomogram Models, Radiomics Model and Clinical Model

Group	Model name	Radiomics model vs. Clinical model	Radiomics model vs.Nomogram model	Nomogram model vs. Clinical model
Training group	LR	＜0.05	0.59	＜0.05
Internal testing group	LR	＜0.05	0.61	＜0.05
External Verification Group	LR	0.10	0.14	＜0.05
Training group	NaiveBayes	＜0.05	0.21	＜0.05
Internal testing group	NaiveBayes	0.06	0.44	＜0.05
External Verification Group	NaiveBayes	0.11	0.07	＜0.05
Training group	SVM	＜0.05	0.69	＜0.05
Internal testing group	SVM	＜0.05	NaN*	＜0.05
External Verification Group	SVM	＜0.05	NaN*	＜0.05
Training group	RandomForest	＜0.05	0.20	＜0.05
Internal testing group	RandomForest	＜0.05	0.19	＜0.05
External Verification Group	RandomForest	＜0.05	0.97	＜0.05
Training group	ExtraTrees	＜0.05	0.05	＜0.05
Internal testing group	ExtraTrees	＜0.05	0.68	＜0.05

External Verification Group	ExtraTrees	＜0.05	0.67	＜0.05
Training group	GradientBoosting	＜0.05	＜0.05	＜0.05
Internal testing group	GradientBoosting	＜0.05	0.71	＜0.05
External Verification Group	GradientBoosting	0.11	0.18	＜0.05
Training group	MLP	＜0.05	0.14	＜0.05
Internal testing group	MLP	＜0.05	0.20	＜0.05
External Verification Group	MLP	＜0.05	0.13	＜0.05

*Regarding the NaN values, they arise because, when combining radiomics and clinical features in the SVM-based opportunistic osteoporosis screening, radiomics features dominate the model performance, while clinical features contribute negligibly. As a result, the Nomogram performs nearly identically to the radiomics model, leading to undefined (NaN) values during comparison. All tables have been reformatted for consistency.
